# Dysmenorrhea of Nasal Origin—With Report of a Case

**Published:** 1912-02

**Authors:** Thos. F. Taylor

**Affiliations:** Tuskegee, Ala.


### DYSMENORRHEA OF NASAL ORIGIN—WITH REPORT OF A CASE.

By Thos. F. Taylor, M. D., Tuskegee, Ala.

Dysmenorrhea has been classed under a number of different heads with reference to its etiology, the main symptom in all being pain in the lower abdomen. Much has been done by surgical means for the relief of this suffering, but occasionally cases are met with which defy all attempts of the surgeon as well as the whole armamentarium of Materia-Medica. It has become customary to refer all nervous manifestations of women that cannot be directly accounted for, to the genital organs, and the patient herself, partly through the intimations of her physician and partly through her natural inclinations, has fallen into the same routine. Especially is this true when there is any accompanying disorder of menstruation.

I am convinced that in many instances we have done actual harm in our ardent attempts toward the radical correction of this disorder. Not a few have suffered repeated curettments, dilatations, and even the loss of an ovary and tube, without the slightest amelioration of the trouble. The next step then being a resort to narcotics, and ultimately landing under the care of the neurologist. The ovary, exercising as it does, such a powerful influence over the nervous system, I am persuaded that we should be slow to remove it unless it is shown, beyond a reasonable doubt, to be the offending member. The mere fact then, that we have not been able to relieve this unfortunate class of sufferers by radical means directed to the genital organs themselves, should lead us to search for the cause, as a reflex condition, in some other region of the body.

The object of this short paper is to call attention to a form of Dysmenorrhea that is barely mentioned in text-books, and, until of late years, seldom seen in journals.

The term "Nasal Dysmenorrhea" originated with Wilhelm Fliess, a laryngologist, in 1897. In his article at that time he gave a series of one hundred or more cures effected by nasal treatment.

At each menstrual period there is a congestion not only in the pelvic organs but in the mucous membranes in other portions of the body. Fliess demonstrated that the mucous lining of the nose was most susceptible to this influence, and located what have since been termed "genital spots"—most marked on the anterior end of the inf-turbinate bone and a corresponding surface on the septum. These areas were shown to be markedly swollen, red and sensitive during the menstrual period, and these symptoms subsided afterwards.

The fact is known to us all that there exists a very intimate connection between the sexual organs and the sense of smell. This is demonstrated in the lower animals during the so-called season of "heat." The anatomy of this condition is difficult to explain, but it is undoubtedly through the sympathetic nervous system, connected with the mucous membrane of the nose by the deep petrosal nerve of Meckle's ganglion, as suggested by Brettauer.

My attention was first attracted to this condition by an article appearing in the American Journal of Obstetrics and Diseases of Women and Children, by Jos. Brettauer, of New York, to whom I acknowledge all credit for what little I have learned on the subject. In this article he sets forths the theory of Fliess, and reports a number of cases he has relieved by this form of treatment.

The symptoms found will be about as follows: Beginning either a few days before, during, or after menstruation, a most intense sick headache, severe pain in back and inguinal regions, accompanied by uncontrollable nausea and vomiting. These patients can retain absolutely nothing by mouth, consequently relief is difficult to obtain except by hypodermatic treatment, and this is but temporary duration. The most annoying symptoms are the headache and extreme nausea. Hot and cold applications are of no avail. Gastric lavage and sinapisms to stomach affording no relief.

In the method outlined by Fliess he includes all cases as nasal that are influenced by the so-called cocaine experiment, which consists in the application of a 20% sol. cocaine to mucous membrane of nasal septum and inf. turbinate bone. This affording almost instantaneous relief from the nausea and headache. This is considered diagnostic, and as soon as practicable, the nose should receive the necessary attention, either through cauterization or removal of growths surgically, as the case may require.

The case that I have to report is a nullipara of about thirty years of age—married ten years—has menstruated normally every twenty-six days up to about three years ago, when she began to have a little trouble at each period. She gives a history of constipation, and during March, 1908, was operated on for appendicitis, at which time an ovary was found to be cystic, and was removed, together with a dilatation and curettage. The dilatation and curettage was done, thinking the menstrual disorder would be relieved. There is nothing in the family history to throw much light on the subject. Instead of being relieved by the oophorectomy, dilatation and curettage, her condition gradually grew worse. The patient having a gastroptosis, it was thought for a while that the stomach was the organ of offense. but the fact that the trouble was always coincident with the menstrual period, pointed strongly to the sexual system. Each month she would be confined to bed from two to eight days. In June, 1911, her condition became critical. The nausea and headache were so severe she was unable to retain medicine or nourishment by mouth, and the resulting exhaustion amounted to almost coma. After a short convalescence she was again curetted, but the July and August periods followed the same routine. During the August period I tried Fliess's cocaine experiment, using first a 4½ sol., without much effect, and the following morning the full 20% strength, which resulted in almost instantaneous relief. Deeming this of some importance, I referred her to a rhinologist of note, who discovered a markedly deviated septum on the left side, covered with hypertrophied mucous membrane, almost closing the left nostril. He did a submucous resection of the septum under cocaine anaesthesia. Following the operation she was dreadfully nauseated, which I ascribed to the mechanical irritation, and took it to be of good omen. At the September period the nose had not healed and was still swollen, and she seemed not to be relieved, but since that time she has passed through four periods with slight inconvenience, not having remained in bed a single day. A second operation, consisting in the removal of the Inf. turbinate, will be done soon, after which I feel very much encouraged that all trouble will be eliminated.

In conclusion, I want to say that I make no extravagant claims for this method of treatment, and can say nothing yet as to its permanent benefits, but merely make this as a preliminary report, in the hope that it may be tried more extensively; and who knows but that it may save many a sufferer the unwilling sacrifice of more important organs.

---

*Discussion*—

Dr. H. P. Cole, Mobile, thanked Dr. Taylor for this paper, as it brought out an interesting phase of this trouble, and he hoped to hear more on the subject when Dr. Taylor continued his paper at some future meeting.

Dr. Wm. Wade Harper, Selma, asked that Dr. Taylor make report at the next meeting of his cases, and the result of further research along this line.

